# Supplementary material for: Identification of photoperiod-induced specific miRNAs in the adrenal glands of Sunite sheep (Ovis aries)
Source: Front Vet Sci. 2022 Jul 22;9:888207. doi: 10.3389/fvets.2022.888207 (PMC9354845; doi:10.3389/fvets.2022.888207)
Supplement: Supplementary file 1 [file Data_Sheet_1.ZIP › Supplementary file/miRDeep2 file.zip!/miRDeep2 file/Novel_Structure/LP42A2/Novel_700.pdf]

Provisional ID	:	NC_019483.2_36815
Score total	:	2.6
Score for star read(s)	:	-1.3
Score for read counts	:	0
Score for mfe	:	2.3
Score for randfold	:	1.6
Score for cons. seed	:	
Total read count	:	23
Mature read count	:	23
Loop read count	:	0
Star read count	:	0

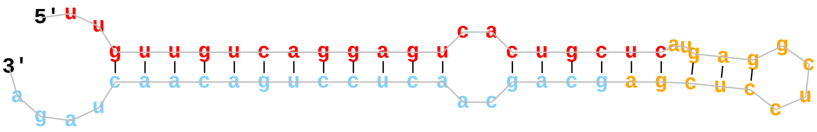

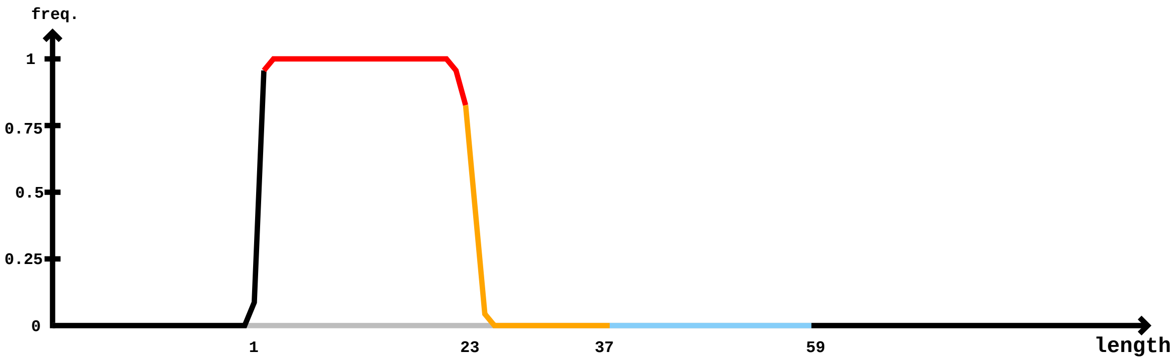

Mature Star

```
5'- gacgauguaggcucacccacuuguugucaggagucacugcucaugaggcuccucgagcagcaacuccugacaacuagaagggagggcagagcaucagcgcagccccugugga  -3'    exp
    ...(((((..((((.(((.((.((((((((((((..((((((..(((....)))))))))..)))))))))))).))..))).))))...)))))..(((((...)))))..    reads      mm         sample
    ...................cuuguugucaggagucacugc........................................................................    1          0          seq
    ...................cuuguugucaggagucacugcu.......................................................................    1          0          seq
    ....................uuguugucaggagucacugcu.......................................................................    2          0          seq
    ....................uuguugucaggagucacugcuc......................................................................    16         0          seq
    ....................uuguugucaggagucacugcuca.....................................................................    2          0          seq
    .....................uguugucaggagucacugcucau....................................................................    1          0          seq
```
